# A wavelet based time frequency analysis of electromyograms to group steps of runners into clusters that contain similar muscle activation patterns

**DOI:** 10.1371/journal.pone.0195125

**Published:** 2018-04-18

**Authors:** Vinzenz von Tscharner, Martin Ullrich, Maurice Mohr, Daniel Comaduran Marquez, Benno M. Nigg

**Affiliations:** Faculty of Kinesiology, Human Performance Laboratory, University of Calgary, Calgary, Alberta, Canada; University of Illinois at Urbana-Champaign, UNITED STATES

## Abstract

**Purpose:**

To wavelet transform the electromyograms of the vastii muscles and generate wavelet intensity patterns (WIP) of runners. Test the hypotheses: 1) The WIP of the vastus medialis (VM) and vastus lateralis (VL) of one step are more similar than the WIPs of these two muscles, offset by one step. 2) The WIPs within one muscle differ by having maximal intensities in specific frequency bands and these intensities are not always occurring at the same time after heel strike. 3) The WIPs that were recorded form one muscle for all steps while running can be grouped into clusters with similar WIPs. It is expected that clusters might have distinctly different, cluster specific mean WIPs.

**Methods:**

The EMG of the vastii muscles from at least 1000 steps from twelve runners were recorded using a bipolar current amplifier and yielded WIPs. Based on the weights obtained after a principal component analysis the dissimilarities (1-correlation) between the WIPs were computed. The dissimilarities were submitted to a hierarchical cluster analysis to search for groups of steps with similar WIPs. The clusters formed by random surrogate WIPs were used to determine whether the groups were likely to be created in a non-random manner.

**Results:**

The steps were grouped in clusters showing similar WIPs. The grouping was based on the frequency bands and their timing showing that they represented defining parts of the WIPs. The correlations between the WIPs of the vastii muscles that were recorded during the same step were higher than the correlations of WPIs that were recorded during consecutive steps, indicating the non-randomness of the WIPs.

**Conclusions:**

The spectral power of EMGs while running varies during the stance phase in time and frequency, therefore a time averaged power spectrum cannot reflect the timing of events that occur while running. It seems likely that there might be a set of predefined patterns that are used upon demand to stabilize the movement.

## Introduction

### Relating electric muscle activity to muscle fiber contractile force generation

Walking and running require dynamic motor control to produce the movement and maintain stability. Surface electromyograms (EMGs) allow only a limited insight in how the muscles work but they are one of the best non-invasive signals to monitor neuromuscular control of the motor task. With respect to timing of muscle activity events one always has to consider that because of the electromechanical delay (86 ms [1] and 25.9 ms [2]) the actual force production occurs much later than the observed peak intensity of an EMG. Although the reported values for electromechanical delay vary substantially, differences in the time of occurrence in peak EMG intensity will most likely be reflected in differences in the time when the force is generated unless one has a very mixed set of fiber types [3].

### How physiological properties relate to the EMG power spectrum

There are diverse physiological properties that affect the power spectra. The muscle fibre contractile force is known to decay with fatigue because of the inability of a muscle to remain sufficiently active. It is common practice to attribute the fall in mean power frequency of the EMG during fatigue to a proportionate fall in conduction velocity of the motor unit (MU) action potentials [4]. However it is still possible that other factors such as de- and recruitment of fibres and change in motor unit firing rates contribute to the fall in mean power frequency during fatigue. A critical alteration of the power spectrum at low frequencies seems to occur because of synchronization of the MU firings [5] and publications that deal with mean and median frequency as indicators of fatigue do not show the spectra and may therefore be unaware of that low frequency modulation. During explosive contractions the concept of mean frequency seems also to fail, for whatever reason [6]. Furthermore it is likely that different populations of MUs can be recruited during dynamic and locomotor tasks. In previous studies, it was hypothesised that the higher-threshold units would contribute higher-frequency components to the EMG spectra due to their faster conduction velocities [7][8]. Morphological variables (fibre proportions and fibre areas) influenced the short term mean frequency–force relationship in vastus lateralis [9]. However, whether different fiber types would be detectable because of their spectral differences was debated in point counterpoint articles [10] [11]. This short introduction shows that it is essential to improve our understanding of the spectral properties of the surface EMG and their physiological causes.

### Time frequency analysis of EMGs

It has been suggested that the time and frequency analysis can shed some light on these questions. The time frequency analysis based on nonlinearly scaled wavelet allows extracting the power of the EMG resolved in time and frequency [12] [13]. Normally the square root of the power, the intensity of the EMG is displayed in a wavelet intensity pattern (WIP). In these WIPs one can observe at what time and at which frequency the intensity of the EMG occurs. This wavelet transform is very flexible and allows to select time and frequency resolutions that can optimally resolve the properties in the lower frequency range (10 to 250Hz). However, the WIPs are very variable and average patterns have often been used to show, for instance, spectral changes that can be interpreted to determine the effort stage of runners [14] or motor strategy patterns of diabetic neuropathic individuals while walking [15]. These promising results showed that the relevant spectral information needs a time frequency analysis to be resolved.

### Assumptions and hypotheses

In the present study we are interested in how the individual WIPs change while running, and our underlying assumption is that the WIPs are non-random. One can expect that during gait the WIPs have some similarities, after all they should reflect the muscle activity that produces and controls the limb movement of similar steps. However, the muscle activity also has to be slightly modulated for each step to contribute to the stability of gait and the necessary variations may be the reason that WIPs recorded for various steps are highly variable. It is therefore the purpose of this paper to study the properties of this variability and investigate whether the wavelet based approach can be used to detect some regularity in the WIPs. Three hypotheses are tested:

1. The correlation between the WIPs of the vastus medialis (VM) and the vastus lateralis (VL) yields a measure of similarity. It is hypothesized that the WIPs obtained from VM and VL that were recorded during the same step will be similar, whereas the WIP obtained from VM and the WIP obtained from VL of the previous or consecutive step would be less similar. In other words, there is a synergistic activation of the vastii muscles during one step that results in similar WIPs for the VM and VL.
2. The WIPs within one muscle differ by having maximal intensities in specific frequency bands and these maximal intensities are not always occurring at the same time after foot strike.
3. During running, WIPs obtained from each of the vastii muscles can be grouped into clusters with steps that were controlled by similar WIPs. The grouping is based on both, the frequency aspects and the timing of the EMG intensity. It is expected that clusters might have distinctly different, cluster specific mean WIPs. It is hypothesised that the median cluster size of the measured pattern is statistically significantly larger than the median cluster size of randomly generated surrogate WIPs. In that case, a limited number of WIPs that were obtained during the running trial are distributed in larger clusters whereas smaller clusters are expected from randomly generated surrogate WIPs.

Hypothesis 1) might be a consequence of the clustering of individual MUs observed in our previous work [16] and that substantial force can only be produced if MUs action potentials cluster because of a common synaptic input to motor neurons [17]. Clustering means that the motor unit action potentials (MUAP) arrive at almost the same time irrespective of the reason for it. Clustering of MUAP can also happen for other reasons than a common synaptic input, for instance because of synchronization of MU [18] a process more precisely defined as classical synchronization [17]. The power of the EMG is most likely averaging out fluctuations caused by single MUs and thus represent a quantity that is related to force production and therefore reflects part of the process that stabilizes the knee joint. If the higher similarity can be proven then one can conclude that the variation of the WIP have a non-random, step specific intensity distribution.

Hypothesis 2) is based on the assumption that there are specific frequency bands that can be assigned to how the EMG is generated e.g. whether various fiber types are activated or if clustering of MUs generate low frequency components. If this hypothesis is correct then one can no longer work with the concept that an EMG during a repetitive dynamic motor task has a single characteristic power spectrum that can be represented by a mean or median frequency only.

Hypothesis 3) might yield a first indication that the WIPs are not caused by random modulations that lead to a mean WIP but its acceptance would rather suggest that humans select from, or mix a set of predefined muscle activation patterns to stabilize the steps while running. This should allow us, in the sense of a pilot project, to decide whether WIPs could, in the future, be analyzed by a pattern recognition approach to study what alters the predefined patterns and their correlations.

## Methods

### Participants and experimental procedure

Twelve healthy, recreationally active, male participants (age: 26 ± 3 years, height: 175 ± 7 cm, weight: 71 ± 6 kg) volunteered and gave their written informed consent to participate in this study. Ethical approval for this research study involving human participants was obtained from the University of Calgary’s Conjoint Health Research Ethics Board, in spirit of the Helsinki Declaration. The participants were asked to run on a treadmill (Quinton Q55, Mortara Instrument Inc., Milwaukee, WI, USA) at a speed of 6.5 mph (10.5 km/h, 2.9 m/s) for a time of 15 minutes to obtain at least 1000 steps per participant.

### EMG signal recording

In order to obtain EMG currents from VM and VL, the skin surface above the muscles was shaved, slightly abraded with sand paper and cleaned with alcohol wipes to ensure high signal conductivity. Bipolar Ag-AgCl electrodes (Norotrode Myotronics-Noromed Inc., US) were placed over the muscle bellies of VM and VL according to electrode locations recommended in SENIAM guidelines [19]. EMG currents were recorded using bipolar version of a previously described monopolar current amplifier [20]. The study was duplicated using a traditional potential amplifier but to keep the manuscript short, only the detailed results of the new current amplifier were reported because the results with the potential amplifier deviated only marginally. The signals were then electronically band-pass filtered between cut-off frequencies of 10Hz (4^th^ order Butterworth filter) and 1000Hz (2^nd^ order Butterworth filter). The amplification was set to optimally use most of the dynamic range of the A/D converter.

### Signal processing and computation of WIPs

The following signal processing was automated and no parameters had to be set for individual participants. All steps of a trial were used and no visual exclusions were made that could bias the results. Foot strike was automatically detected from the accelerometer signal. EMG periods were selected during time windows between 120ms prior to foot strike and 200ms after foot strike, when the muscle was active. These EMGs were wavelet transformed using the equations quoted in [12] [13]. There were 20 symmetric, non-orthogonal Cauchy wavelets with the following center frequencies (2.5, 5, 8.2, 13, 19, 25, 35, 44, 55, 65, 80, 94, 107, 125, 142, 160, 180, 202, 222, 247Hz). These wavelets are discrete in frequency using 20 distinct wavelets (discrete wavelet transform in frequency). The wavelet transform was almost continuous in time using fixed time intervals of dt = 6.67 ms. The wavelets were selected because of their symmetrical shape, thus they do not introduce time shifts of the observed intensities. The intensities obtained by the wavelet transform were resampled in time to yield 48 time points whereby foot strike was at point 18. The WIP thus represent the intensity at 20 frequencies and 48 time points and are represented by a pattern-vector with a dimensionality of 960. Because the low frequency part of the power spectrum might be strongly modulated by MUAP that cluster there are suggestions that recommend to focus on higher frequency ranges [21]. All wavelet patterns for each vastii muscle were normalized to a single normalization factor that was obtained in the frequency range between 107Hz and 247Hz, where the clustering of MU is expected to have a minimal effect. Specifically, the WIPs were averaged across all steps, the intensities for each wavelet were averaged over time, and the sum of the mean intensities in the frequency bands between 107Hz and 247Hz yielded the normalization factor.

To represent the WIP in a lower dimensional space, pattern-vectors from all steps (N > 1000) from VM and VL were combined in one matrix (M_raw). The size of M-raw was 2N x 960. The mean of all patterns (M_mean) was subtracted and a principal component analysis was applied yielding PC-vectors. Only a subset of PC-vectors, those that explain 99% of the variability were retained. The M_mean was then projected onto the subset of PC-vectors to obtain the part of M_mean that can be reconstructed from these PC-vectors. The residual of M_mean is the difference M_mean–M_mean_reconstructed. The residual vector (PC-residual) was normalized to 1 and added as the first vector to the subset of PC-vectors. Together they form the base-vectors of the new, lower dimensional vector space that can represent all WIPs inclusive the mean WIP. The dimensionality of this space is called new dimensionality (nd). The weights (W_VM and W_VL) representing the WIP of the VM and VL muscle were obtained projecting the pattern vectors of M_raw onto these base vectors. Each weight of W_VM or W_VL has a mean (W_VM_mean, W_VL_mean) and a standard deviation (W_VM_std, W_VL_std). This is called a weight distribution of the raw WIPs. Each WIP can be reconstructed using a linear combination of the weights (w_i_) multiplied by the base-vectors (PC_i_).

$$WIP=\sum_{i=1}^{nd}w_{i}∙{PC}_{i}$$

Surrogate WIPs were generated using the same base vectors combined with random weights (w_i_) drawn randomly from the weight distribution that was obtained from the raw WIPs. If the raw WIPs were represented by random mixtures of weights drawn from the weight distribution, then the clustering of a set of surrogate patterns would yield almost the same result compared to the clustering obtained for the raw WIPs. The surrogates were therefore used to test whether the raw WIPs could be explained using a random superposition of weights.

### Similarity of WIPs

To assess the similarity of WIPs the correlation between the weights representing the WIPs was computed. First the correlation of WIPs of VM and VL that were obtained during the same step were computed. Thereafter, the correlation between a WIP from VM and a WIP from VL of the consecutive step was computed. The paired differences between the two correlations were computed for all steps. The mean and the standard error of the paired differences were computed. A student’s t-test was used to assess whether the paired differences were significant (hypothesis 1).

### Cluster analysis

The cluster analysis was performed individually for the VM and VL muscles. The correlations between all WIPs of one muscle that were obtained for all steps were computed. For the purpose of finding clusters, it is advantageous to use a measure of dissimilarity, which was defined as 1-correlation. The dissimilarity was used to form an agglomerative hierarchical cluster tree by the Matlab linkage function using the furthest distance method, which returns a matrix Z that encodes a tree of hierarchical clusters of the rows of the matrix containing the dissimilarities. A dendrogram plot was used to display the tree of hierarchical clusters. To construct agglomerative clusters from linkages the Matlab cluster function was used. The cut-off value was the mean dissimilarity plus one standard deviation and the criterion required by the Matlab function was “distance”. As a result, one obtains a number of clusters, each characterised by an index (k). The WIP of each step is then assigned to the cluster_k_. Thus, each cluster contains the WIPs from multiple steps. The number of WIPs in a cluster is called the cluster size. A histogram was constructed showing the number of clusters that had the same cluster size. The median of the cluster size was computed for the raw WIPs. The higher the median cluster size is, the more steps are in larger clusters. The median cluster size values of surrogate WIPs were computed from twenty-five sets of N surrogate WIPs. A sufficiently large number of sets was needed to accurately compute the average and standard deviation of median cluster sizes. If the median cluster size of the raw WIPs is outside the 95% interval of the surrogate WIP’s median cluster sizes, then one can conclude that there were non-random similarities between the raw WIPs of the original steps in the clusters (hypothesis 3). From all raw WIPs of clusters with cluster sizes above the median cluster size, the two that had the greatest dissimilarity were selected to discuss the main aspects causing the dissimilarity.

## Results

Typical results of individual subjects will be shown first to illustrate the extraction of variables reported in Table 1 and to reveal general properties of WIPs while running. The mean across all subjects of the values in Table 1 obtained from the EMG potential amplifier deviated by less than 4.5% from the values obtained with the current amplifier and details are therefore not reported.

**Table 1:** Results of correlation analysis and hierarchical clustering.

subject#	1	2	3	4	5	6	7	8	9	10	11	12
new dimension of												
PC_vector space	104	101	126	116	117	112	97	122	95	124	122	130
correlation VM/VL,												
mean · 100	83	72	75	82	80	79	84	71	84	65	76	70
correlation VM/VL,												
ste. **·** 100	0.1	0.2	0.1	0.1	0.1	0.2	0.1	0.2	0.1	0.2	0.1	0.2
correlation VM/VL,												
slope · 100000	1.0	3.6	0.0	-6.1	-0.4	1.8	0.7	-0.3	-2.0	7.3	3.7	-0.6
correlation VM/VL,												
p of slope · 100	0.4	0.0	92	0.0	37	0.2	4	57	0.0	0.0	0.0	19
correlation paired												
difference, mean ·100	2.1	0.2	0.7	3.5	2.4	2.1	3.4	1	4.1	0.9	0.6	1.7
correlation paired												
difference, ste. · 100	0.2	0.1	0.2	0.2	0.2	0.2	0.2	0.2	0.2	0.2	0.2	0.2
dissimilarity VM, mean												
of all combinations·100	19	15	17	18	20	20	18	21	19	19	21	24
dissimilarity VM,												
std. · 100	5	4.6	4.8	4.5	4.6	5.1	5.1	5.3	5.8	7.3	5.1	5.4
cut-off for hierarchical												
clustering, VM · 100	24	19	22	22	25	25	23	26	24	26	26	29
number of clusters, VM	65	50	56	62	70	56	56	57	44	20	60	56
max. number of steps												
in a cluster, VM	54	125	84	54	57	89	78	70	69	219	60	74
median of number of												
clusters, VM	17	17	18	18	13	16	19	15	26	30	20	19
number of clusters,												
VM surr	113	78	86	111	123	118	115	99	78	75	114	130
max. number of steps												
in a cluster, VM surr	82	55	81	50	39	47	76	61	71	284	60	44
median of number of												
clusters, VM surr	7	10	9	7	7	5	6	7	11	3	8	5
dissimilarity VL, mean												
of all combinations·100	18	15	19	19	20	17	18	20	19	19	20	22
dissimilarity VL,												
std.·100	5	4.3	4.4	5.8	5.2	5.2	5.2	4.8	5.7	4.3	5.1	5.1
cut-off for hierarchical												
clustering, VL · 100	23	19	24	25	25	22	23	25	25	23	25	27
number of clusters, VL	59	60	70	42	54	53	58	61	53	76	62	57
max. number of steps												
in a cluster, VL	81	61	40	101	76	70	95	43	116	39	72	50
median of number of												
clusters, VL	17	17	16	20	20	17	16	14	18	15	17	18
number of clusters,												
VL surr	107	85	116	74	102	76	110	99	105	114	102	119
max. number of steps												
in a cluster, VL surr	69	47	51	89	87	67	92	38	136	50	79	73
median of number of												
clusters, VL surr	6	9	7	9	6	9	7	7	6	8	9	5

**Fig 1:**
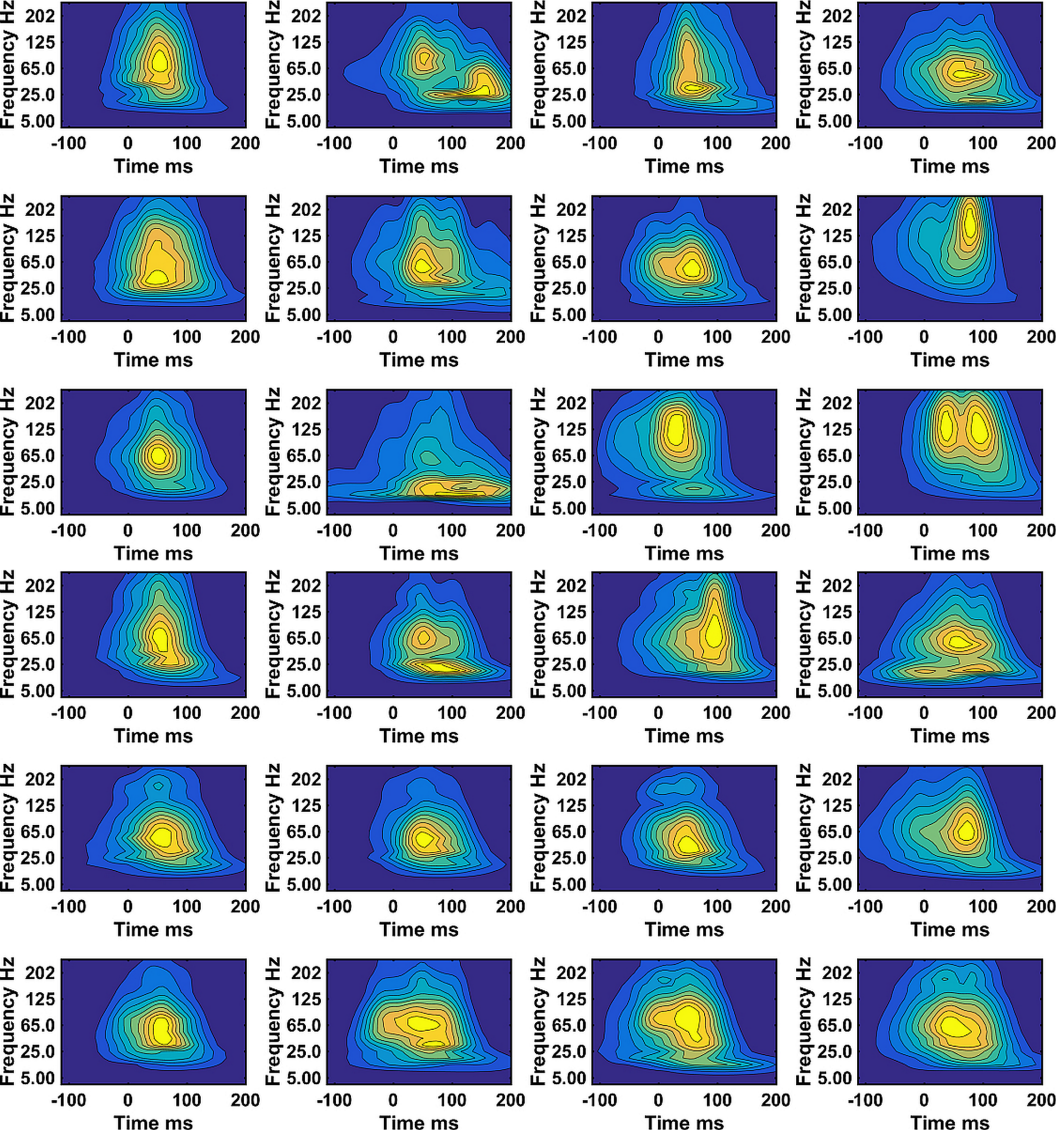
Mean wavelet intensity patterns (WIPs). a) VM and b) VL for all steps from 12 subjects. The subject # is ascending from left to right and top to bottom. The intensities were normalized to the mean intensity in the frequency range covered by the wavelets with center frequencies 107 to 247Hz.

Between 1209 to1374 steps per participant were used to compute the WIPs representing the muscle activity. The mean over all steps, of the WIPs of VM and VL, for 12 subjects show subject specific results indicating the probability that the muscle will be activated at a certain frequency and time (Fig 1). Individual subjects show WIPs with specific characteristics. Most subjects show a high intensity in a wide frequency band, however, subject 8, 11 and 12 show distinctly higher frequencies for the VM muscle. There are two other frequency bands, one around 40Hz and one at 25Hz or below that significantly contribute to the WIPs either at a different time or just forming a distinct separate maximal intensity at a lower frequency (subject 2 and 4). The WIP seem skewed at low frequencies towards times further away from foot strike.

**Fig 2:**
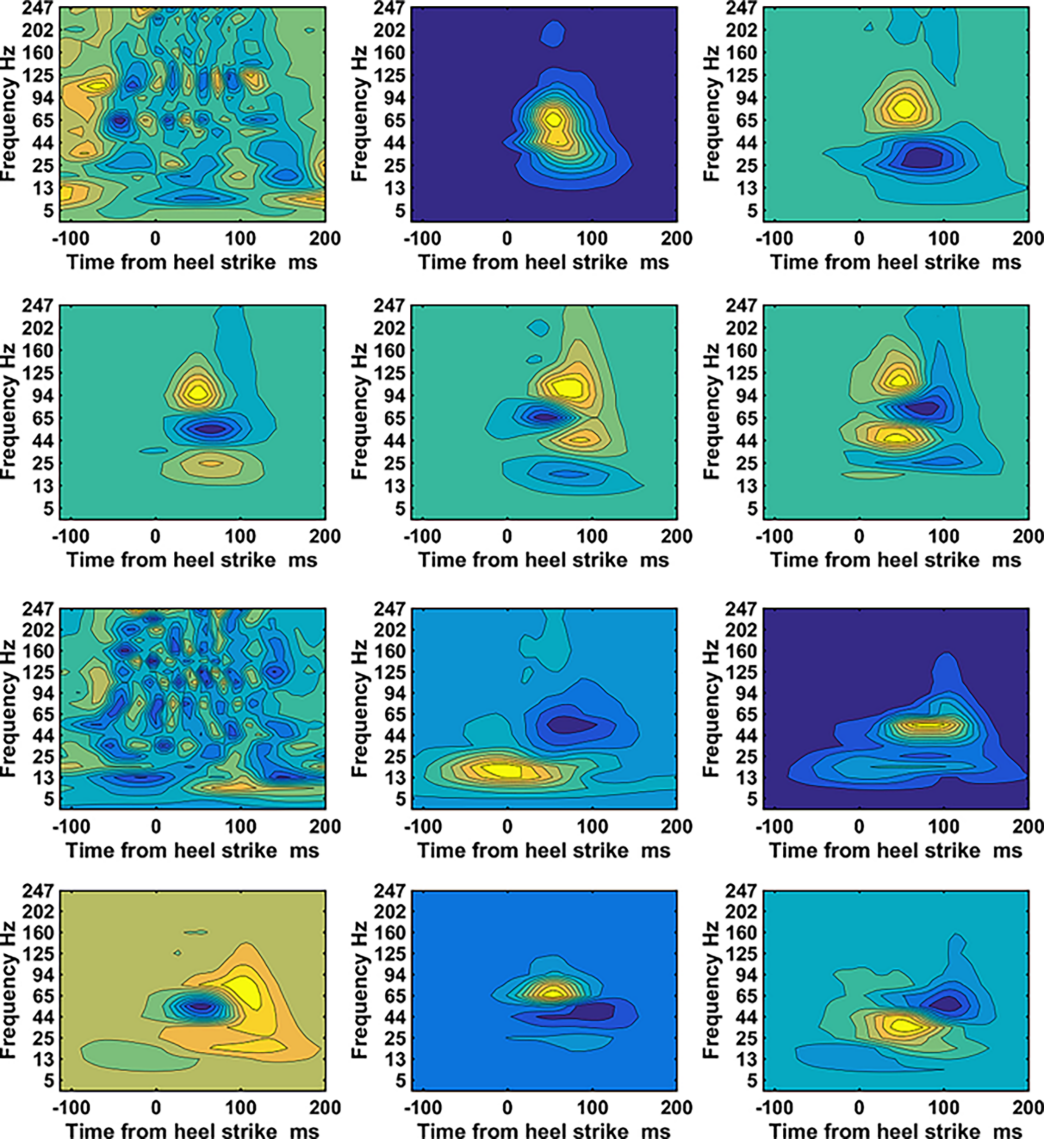
WIPs of the PC vectors obtained from both vastii muscles. a) subject#1 and b) subject#4. Top left is the WIP of the PC-residual vector of the mean, it is needed to be able to reconstruct the mean. The WIP of the residual vector is followed by the WIP of the first five PC-vectors. The maximal positive value is indicated in bright yellow and the maximal negative value is indicated in dark blue. The norm is 1 for all vectors. All PC-vectors and the PC- residual vector are orthogonal to one another. The number of PC-vectors that explain 99% of the variability together with the residual form the new base for the patterns and has the new dimensionality, nd (Table 1).

**Fig 3:**
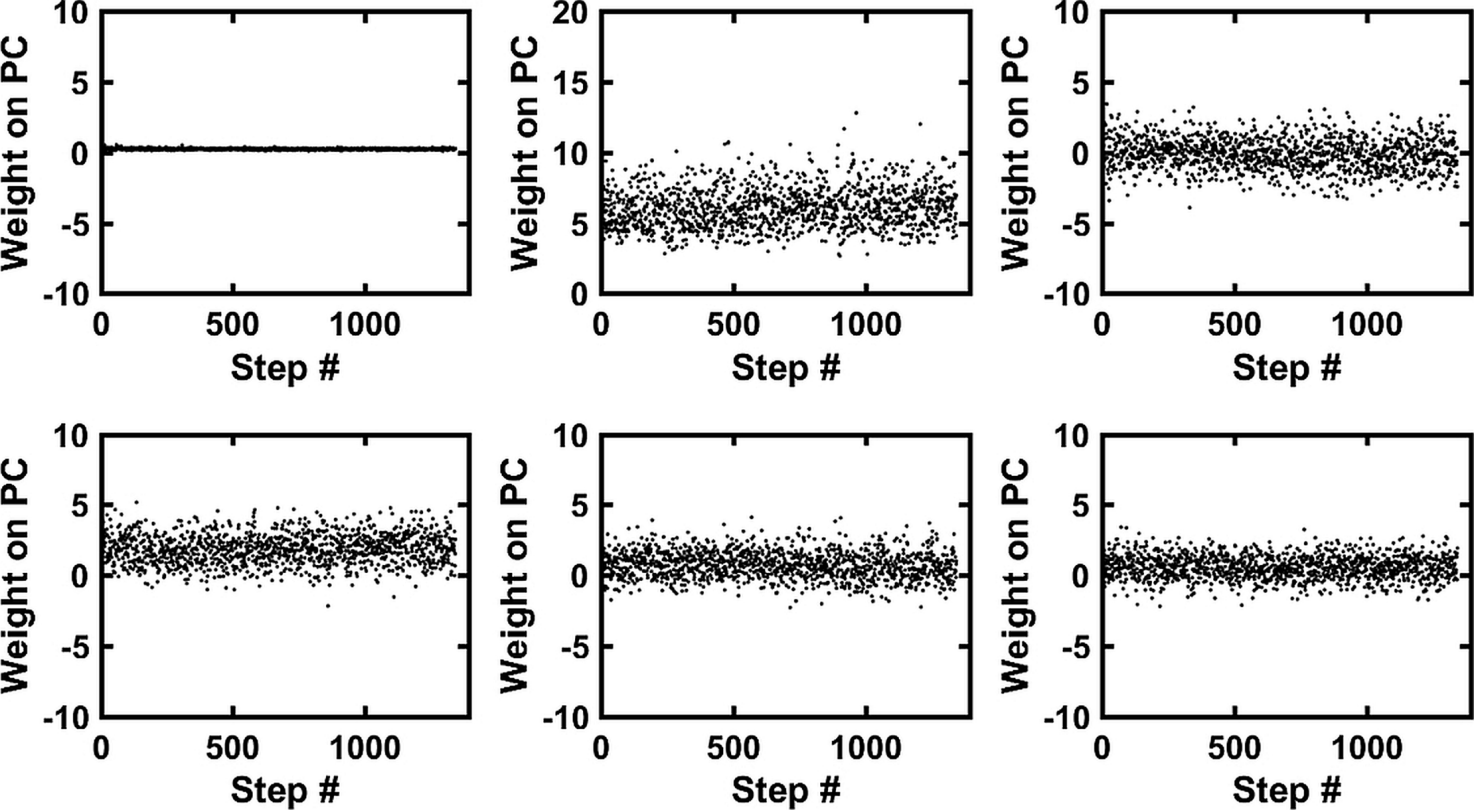
The weights from the PC analysis. The weights of subject#1 obtained by projecting the WIP pattern vectors of the steps onto the PC-vectors. The weights on the PC-residual (Top left) are very small but significantly contribute to the WIPs. The trends of the weights, if present, show an adaption over time of that WIP.

The dimensionality of a WIP is 960 and was reduced to a new dimensionality of, on average 113, by the principle component analysis. The WIP of the first five PC-vectors obtained from both vastii muscles are shown for subject 1 together with the WIP of the PC-residual (Fig 2). WIPs of PC-vectors show patterns that contribute as a whole to the final WIP of one step. The contribution is indicated by the weight factor. For this subject the PC-vectors most distinctly differentiate the frequency bands mentioned in Fig 1 above. The WIP of the first PC-vector indicates a clear dominant band at about 80Hz it also shows a distinct sideband at 40Hz and a very weak but distinct band at 200Hz. This WIP contributes most to the WIP of the step, it gets modulated by the contributions (weights) of the WIPs of the higher order PC-vectors. The PC-vector#2 shows that intensities at frequencies at 80Hz thus slightly above 65Hz represent a trade-off for frequencies at around 25Hz. Finally the WIP of PC-vector#4 contributes to 4 frequency bands. Thus one can subdivide the frequencies into 4 frequency bands, one below 25Hz then the 40Hz band in the range (34Hz to 53Hz) followed by the 80Hz band in the range (65Hz to 94Hz) and a high frequency band above 107Hz. The PC-vectors show that these frequencies do not necessarily occur at the same time during gait. Of interest is also the WIP of the residual-vector. Its contributing weight is small compared to the weights of the five first PC-vectors (Fig 3). However, specifically in the 80Hz band and 120Hz range it reveals a periodicity. Because it is the residual of the mean, it describes an aspect that is common to the mean and thus to the WIP of all steps.

The first few WIPs of the PC-vectors reveal the dominant frequency bands that are contributing to the WIPs. The WIP of the PC-vector#1 contributes with positive weights and it contributes the highest variability (about 20% of the total variance) (Fig 3). The weights of the PC-vector#2 contributes about 10% to the total variance. Thus, it indicates a step to step variability of the spectrum of the EMG. The step to step variability of the first 5 PC-vectors show that the different frequency bands do not contribute equally to each step. On average about 110 PC-vectors are needed to explain 99% of the variability of the WIPs whereby the first 5 explain already about 50%. PC-vector#6 only contributes 3% and the higher order ones even less.

The mean weights over all steps for PC-vector#1 and #3 are 5.9 and 1.8 and they contribute most to the mean WIP. For subject#4 the mean weights over all steps for PC-vector#2 and #4 are 8.2 and 3.6 and they contribute most to the mean WIP. These PC-vectors represent the frequency bands that contribute most significantly and in a correlated way to the mean wavelet spectrum. In the same way one can interpret the contributions of the higher order PC-vectors either to the variability or to the mean WIP.

The mean absolute correlations between the weights of all possible pairs of PC-vectors were in 86% of the cases not significantly different from zero. The remaining 14% significant absolute correlations were on average very small (0.002). Therefore, we considered the weights of different PC-vectors as being independent, which is a necessary feature that is required for the surrogate WIPs to be part of the same weight distribution than the measured WIPs.

**Fig 4:**
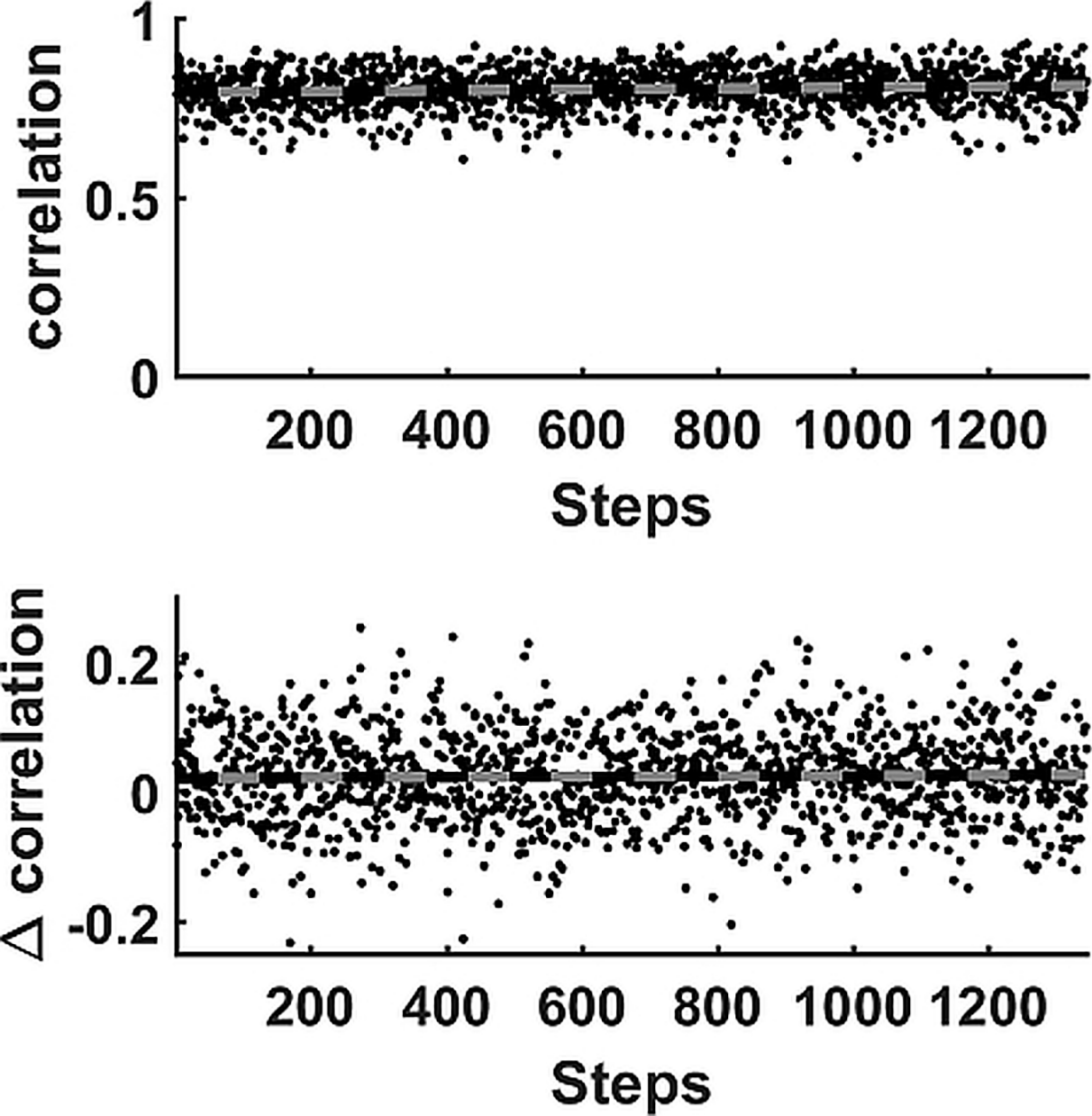
Stepwise correlations. Correlation analysis for subject#10: Top: The correlations where the WIPs were obtained from the VM and VL muscles during the same step. These correlations occasionally show a significant change over time. The computed trend line indicates that the correlation increased systematically while running. Bottom: The paired differences in correlation between the WIPs that were obtained from the VM and VL muscles during the same step and those obtained during consecutive steps. This eliminates the effect of the trend (the trend line shows no significant slope) and allows measuring the small absolute paired differences reported in Table 1.

The weight-vector contains the weights of the projections of the WIPs onto the PC-vectors and thus has a length indicated by new dimension (Table 1). One such vector defines a WIP of one muscle during one step. The correlation of two WIPs can thus be computed for WIP of the VM muscle and WIP of the VL muscle (Fig 4 top). The correlations are normally fairly constant showing either a slight positive (6 subjects) or slightly negative (3 subjects) trend, however the trend for subject#10 was not linear. The result indicates that the correlation between the vastii muscles can adapt while running but we did not observe a systematic variation. Irrespective of the trend, the correlation between the WIP of VM and the WIP of VL of the consecutive step was significantly lower for all but one of the 12 participants than the correlation between WIPs of the same step (Fig 4 bottom) (Table 1). However, the average difference, although highly significant, was on average only 0.019 correlation units and thus sometimes less than the changes caused by the drifts mentioned above.

**Fig 5:**
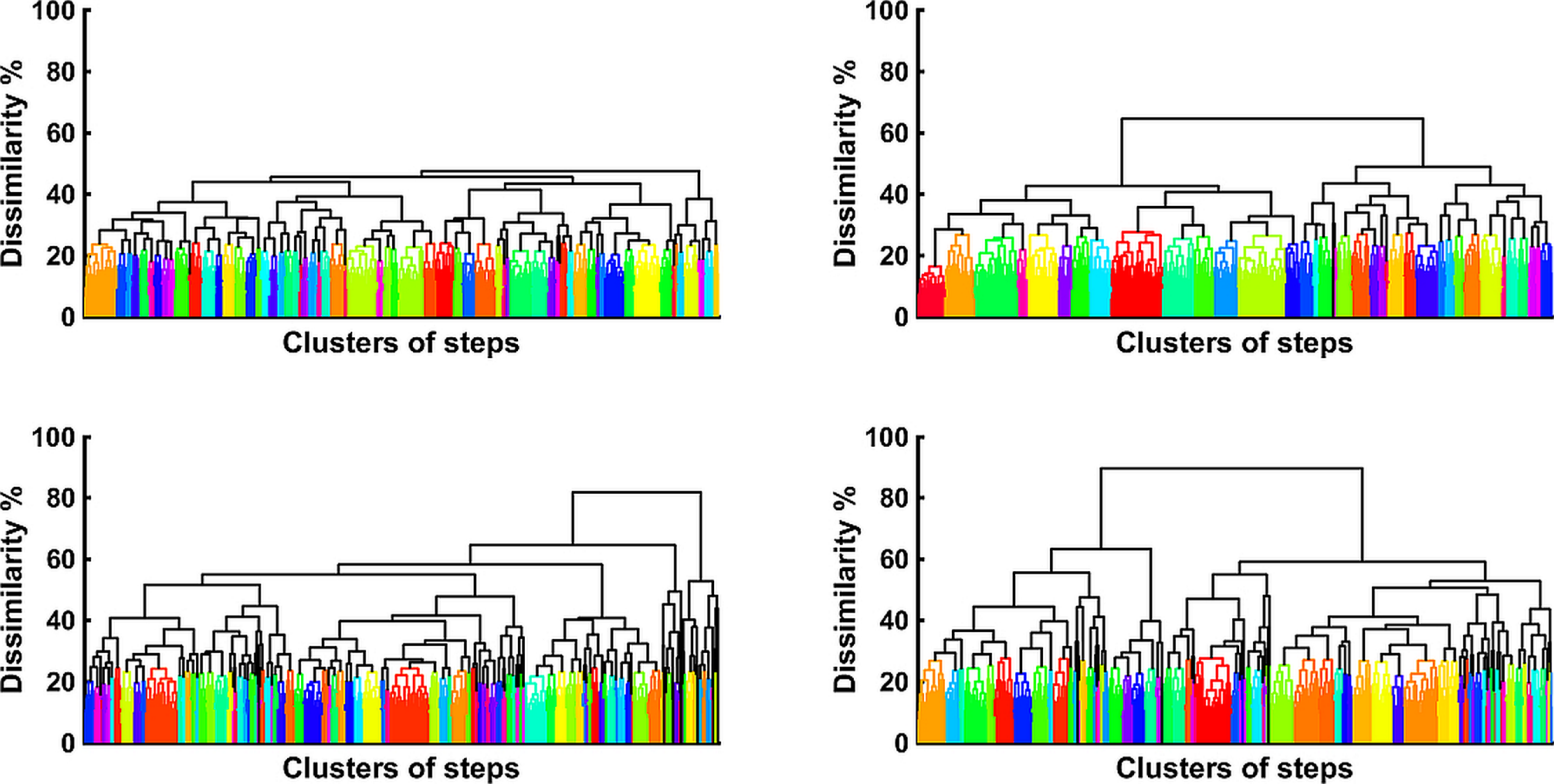
Hierarchical clustering. Hierarchical clustering of the intensity patterns recorded from 1221 steps of subject#4. VM (Top left) and VL (Top right) and for the clustering of surrogate WIPs of VM (Bottom left) and of VL (Bottom right). A high-resolution copy of this figure is provided in the supporting material (S1 Fig).

**Fig 6:**
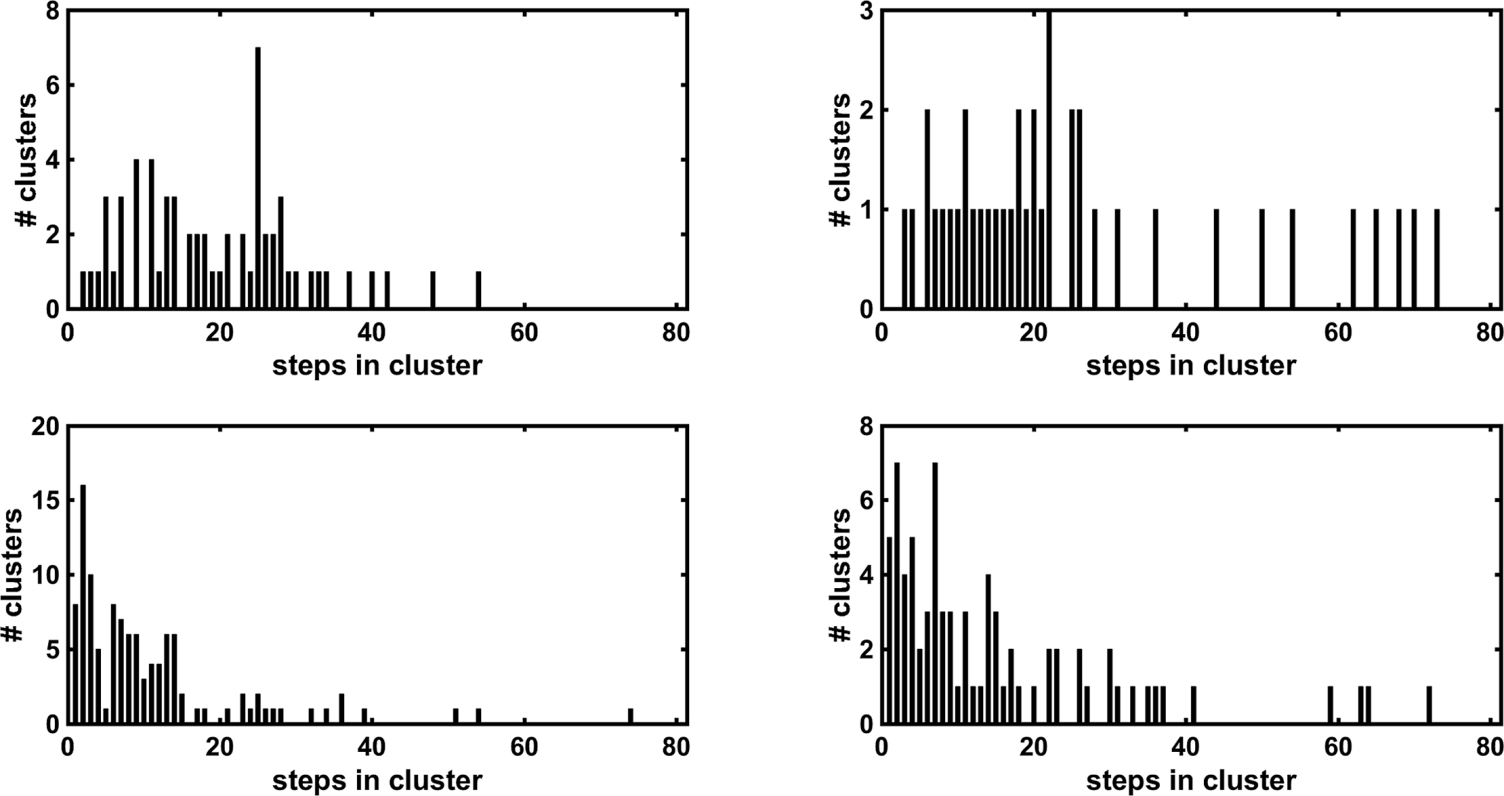
Histograms of cluster sizes. Histograms of subject#4 showing the number of clusters containing a certain number WIPs of steps per cluster. The top graphs show the results for VM and VL whereas the bottom ones the results of the corresponding surrogate WIPs. The surrogate WIP show more clusters with a smaller cluster size (Table 1).

WIPs of all steps of one muscle look very similar and therefore will yield small dissimilarities (1-correlation) among them. The dissimilarities of all possible combinations of WIP of one muscles yielded an average value and a standard deviation (Table 1). About 85% of the dissimilarities of all possible combinations have a dissimilarity value of less than the mean + one standard deviation of all dissimilarities (Table 1). This value was used as a cut-off value for a hierarchical clustering process, which resulted in a subject specific number of clusters between 20 and 70 (Fig 5 left side) (Table 1). The same cut-off value was used for surrogate WIPs and the hierarchical clustering is shown on the right side of Fig 5. The result shows that surrogate WIPs have larger maximal dissimilarity values which is an indication that the non-surrogates have something in common. Each of these clusters contained a number of steps, which determine the cluster size. The numbers of clusters with an equal cluster size are shown as a histogram for both vastii muscles and for their surrogate WIPs (Fig 6). The standard deviation of the median cluster size was 0.87 for the surrogate WIP. According to this standard deviation, the medians of the cluster size are significantly lower for the surrogate WIPs than for the raw WIPs (Table 1). For all subjects the raw WIPs form clusters with more steps per cluster than the surrogate WIPs thus the result is that they were not generated by random combinations of weight factors.

**Fig 7:**
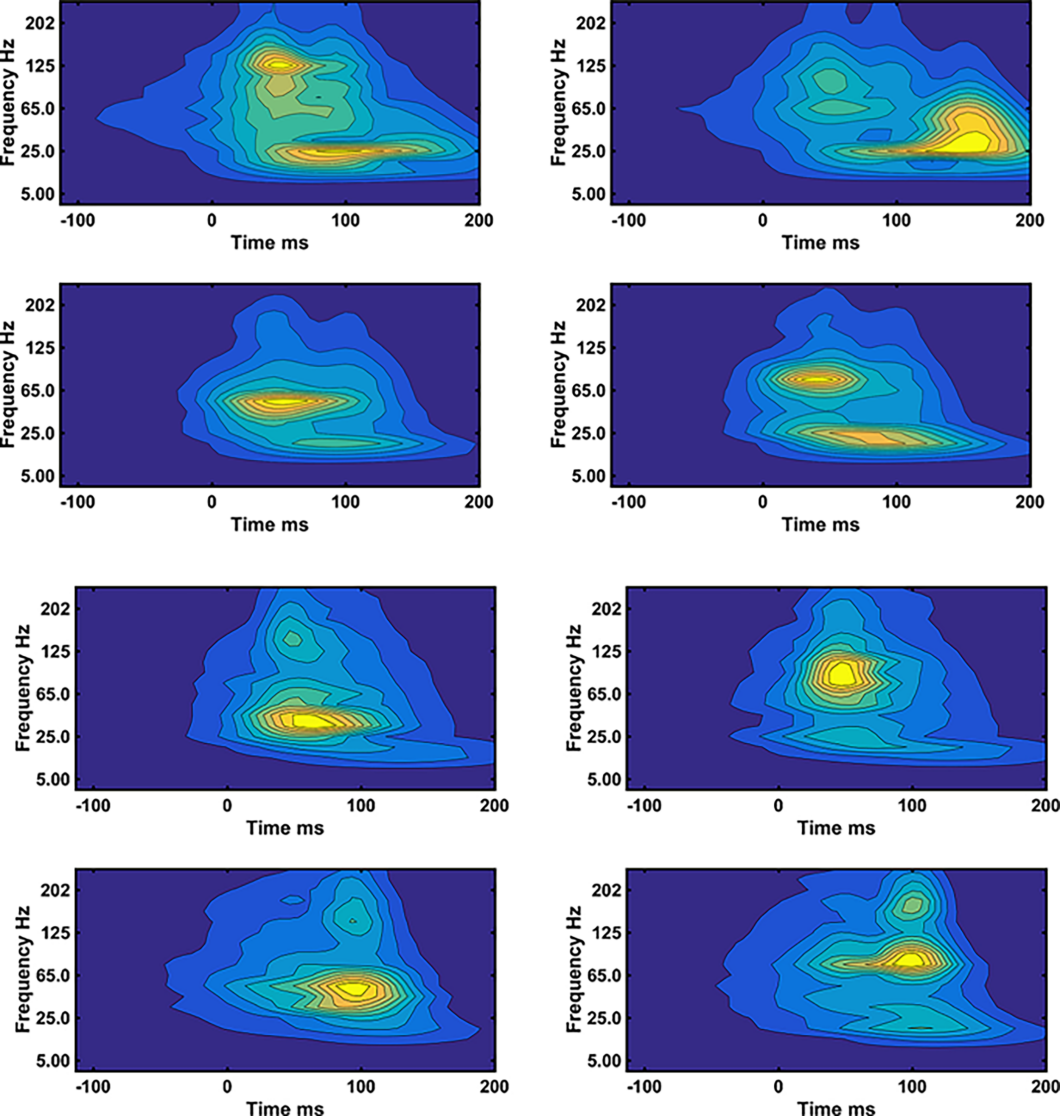
Reconstructed mean WIP. Reconstructed mean WIP of the two clusters (left and right) showing the highest dissimilarity for a) subject#2 b) subject#3. Top for VM, bottom for VL.

One can inspect the mean of all steps in the two clusters that showed the highest dissimilarity (Fig 7). The WIP show that the differences are basically caused by the activity in four relatively distinct frequency bands that get activated individually in the WIPs of different clusters. The high frequency band is located between 107 and 247Hz, the mid frequency band is located around 80Hz (65 to 94Hz), there is a distinct band that centers around 40Hz (between 34Hz and 53Hz) and finally there is a low frequency band that usually appears at 25Hz and below. These frequency bands are the same ones resolved by the PCA analysis. The low frequency band is timewise frequently shifted to the right, to a time range 100 to 200ms after foot strike whereas the other bands are more likely to occur between 20 and 100ms after foot strike. The bands are, however, not necessarily all located at the same time showing that the EMG activity occurs at different times in different frequency bands for different clusters of steps.

## Discussion

The values for the paired difference of correlations between the WIP of the VM and VL for the same step are significantly larger than the correlations between consecutive steps (Table 1). The difference is on average 0.019 ± 0.002 and thus supports the hypothesis that there is synchronization in the sense that the events occur at the same time and frequency in the two vastii muscles at the level of their EMG intensity. This was expected because of the high coherence observed previously for the vastii muscles during squatting [16]. The result is significant although much lower than one would expect when considering how highly coherent the individual MUs were between the vastii muscles. A possible reason might be that the common mode rejection of the amplifier rejected exactly those parts of the signal that were coherent. Therefore the coherence of the MUs will be investigated for running in a future study.

It was hypothesized that WIPs differ by having maximal intensities in specific frequency bands. A first indication can already be seen in the WIPs that were averaged over all N steps (Fig 1). The WIPs of subject 2 and 4 reveal these bands distinctively and one can conclude that there is a low frequency band at 25Hz and below that occurs later in time than the main frequency band that was centered around 80Hz. More distinctively the bands appear in the first five PC-vectors (Fig 2). These PC-vectors represent areas of the pattern that contribute together, thus in a correlated way to the final WIP. These PC-vectors show about four distinct main frequency bands. It is the way how the EMG intensity in these frequency bands is distributed in the mean WIP of clusters of steps, and thus separates the WIPs of groups of steps that further supports the second hypothesis of specific frequency bands (Fig 7). These bands can only be resolved by using wavelets with higher resolution at low frequencies and would be smeared out when the wavelet center frequencies are more widely spaced [12]. The low frequency band at 25Hz and below might be the one reported by van Boxtel for performing static contractions of the frontalis and corrugator muscles [5]. A similar low frequency peak was derived theoretically for “high synchrony 100% excitation” by Yao et al. [22]. However, multiple peaks, as predicted by Pan [23], were not observed. All these models are based on classical synchronization, thus on pulse trains and are therefore most likely not applicable to the observed short bursts of muscle activity of runners. A more likely explanation could be that a series of rapid firings, 2 to 4 pulses at about 15ms inter spike intervals or like-duplets [24] [25] merge to a subject specific, low frequency action potential at the surface of the skin. If the first pulse occurs at the same initialization time than the first pulse of other MUs then the other pulses occur later and the resultant action potential is most likely shifted to later times as observed in Fig 1 for subject 2, 3, 4, 10. This would indicate that the low frequency band would be caused by the clustering of MUAP elicited by these few rapid firings. It may still be elicited by a common synaptic input to motor-neurons [26] because a common synaptic input is necessary to activate the muscle in time intervals as short as those shown in the WIPs.

The frequency band that contains the highest average power is located between 35Hz and 107Hz (Fig 1) and represents the frequency band already reported by Merletti & Lo Conte [27]. It represents the power spectrum of the majority of MUAPs, which is, among other effects, most significantly altered by peripheral fatigue and is sensitive to changes in muscle fiber conduction velocity. However, the PC-vectors shown in Fig 2 indicate that there are at least two frequency bands to be considered in that same range, one around 40Hz and the other one between 60Hz and 107Hz. The 40Hz band was distinctively resolved in PC-vector#3 of subject#1 and can also be seen in the PC-vectors of subject#4 (Fig 2). It was in the 40Hz band where the raw EMG signal showed the highest coherence while squatting [16]. One might thus interpret this band as the one that is caused by superimposed MUAP that widen the summed MUAPs and thus lower the apparent frequency. This would be consistent with the frequency shift of the instantaneous mean frequency, which dropped from about 72Hz to 60Hz during an explosive motor task [6]. Thus, this is an additional effect to the one that caused the 25Hz band. It is worth noticing that on one hand there are clusters of WIPs of steps that all show a dominance of the 40Hz peak without a lot of intensity in the other bands, while on the other hand, there are clusters of WIPs of steps that show practically no intensity at 40Hz (Fig 7). It is not clear why MUAPs would superimpose in one set of steps but not in other sets. After all, it was demonstrated that higher and lower frequency components in the myoelectric spectra can be present when the faster or slower MUs are assumed to be active [7]. Thus part of the 40Hz peak could also be caused by a subset of MUs.

Finally, there is a distinct frequency band between 107Hz and 250Hz (Fig 7). For subject#8, this band becomes the dominant band (Fig 1). In other subjects, there are clusters of steps where this band seems to dominate (Fig 7). The most likely interpretation is that this band represents the fast MUs described by Wakeling and Rozitis [7]. Very little is known about this frequency band. It is always observable and parts of it could therefore also represent spectral aspects of the fine structure of MUAPs. Such fine structures can be the result of the endplate distributions of the MUs.

In summary, the power spectrum of an EMG during a dynamic motor task is a result of multiple processes that each affect the spectrum in different frequency bands.

We already used the result of the hierarchical clustering analysis to discuss the various frequency bands. The comparison of the median cluster size of the raw WIPs with the median cluster size of the surrogate WIPs seem to indicate that the clusters of WIPs are not caused by a random superposition of variable WIPs supporting the third hypothesis. The result rather indicates that there are groups of steps that occur at different time points during the run and have similar WIPs. The mean WPI of the clusters, although numerically separated, looked very similar and only subtle differences discriminates them. Distinctly different patterns can be visualized looking at the most dissimilar ones only (Fig 7). One tentative interpretation could be that the activation strategy of these steps is centrally predefined and that the strategies are activated when needed. Even though, the patterns appear very variable, however, they are not without a distinct structure (Fig 7). It is most likely that this variability is needed for specific adjustments of the movement or the stability during gait. The main limitation of the unsupervised classification of steps is caused by the decision how to set the cut-off value for the dissimilarity. If the cut-off is set too low then the cluster sizes become small, if the cut-off is too high, only a few groups will be isolated and they might not be meaningful. Thus the result cannot be used to decide how many predefined WIPs are used by a certain individual. However, the method clearly shows that there are repetitive WIPs that are distinctly different in various clusters (Fig 7). The differences are located in characteristic frequency bands and thus, it is important to further improve our knowledge of how the physiological properties that control the motor task translate into these frequency bands.

In contrast to the distinct events that were visible in the WIPs of the PC-vectors the PC-residual (Fig 2) could be interpreted as background noise. However, without using a base that contains the PC-residual a correlation analysis was not revealing actual correlations between patterns. Using the base including the PC-residual allows the representation of the actual WIPs by the weights and thus takes care of the fact that each pattern does not have the same contribution from the mean WIP. Subtracting the mean before the analysis corrupts the computation of correlations and dissimilarities. The visual analysis of the details of the WIP of the PC-residual (Fig 2) reveals frequency dependent periodic intensity fluctuations. According to our previous work these periodic oscillations are in phase with the foot strike and start before foot strike, which our current finding confirms. They reflect the Piper rhythm[28]. It seems that it is this basic structure that changes when performing higher effort tasks and allowed classification of effort levels of runners [14].

We are well aware of all the discouraging limitations of surface EMG analysis that are regularly published [29] [30] but tried to proceed in another direction. We also did not consider firing rates and long pulse trains in our discussion, because our muscular events were too short for longer pulse trains to develop. It should be mentioned that when motor unit action potentials overlap part of the signal is removed and the power of the EMG signal drops. The process is known as signal cancelling [31]. However, when motor units strongly overlap, signal enhancements occur and the power increases because of the overlap. More recent models compute the power including signal enhancement and cancellation [32]. Thus, intensities caused by clusters may overshadow the ones from non-clustered MUAPs depending on the degree of overlap. The use of PCA and hierarchical clustering is only one possible way for detecting structures in the WIPs, independent component analysis and Kohonen maps would be other possible choices. The present observations show that it is possible to isolate frequency bands and compare relative intensities and their timing. Over time, one will be able to refine the above interpretations and replications by other laboratories are necessary.

## Supporting information

S1 Dataset Raw EMG. Matlab-file with raw EMG of Vastus Medialis (1^st^ column in each cell) and Vastus Lateralis (2^nd^ column in each cell) for all steps (n > 1000) and all subjects (n = 12).
(MAT)

S1 Fig High-resolution version of Fig 5. Hierarchical clustering of the intensity patterns recorded from 1221 steps of subject # 4. VM (Top left) and VL (Top right) and for 1221 surrogate patterns of VM (Bottom left) and of VL (Bottom right).
(TIF)
