# Supplementary figures and images for: A wavelet based time frequency analysis of electromyograms to group steps of runners into clusters that contain similar muscle activation patterns
Source: PLoS One. 2018 Apr 18;13(4):e0195125. doi: 10.1371/journal.pone.0195125 (PMC5906018; doi:10.1371/journal.pone.0195125)

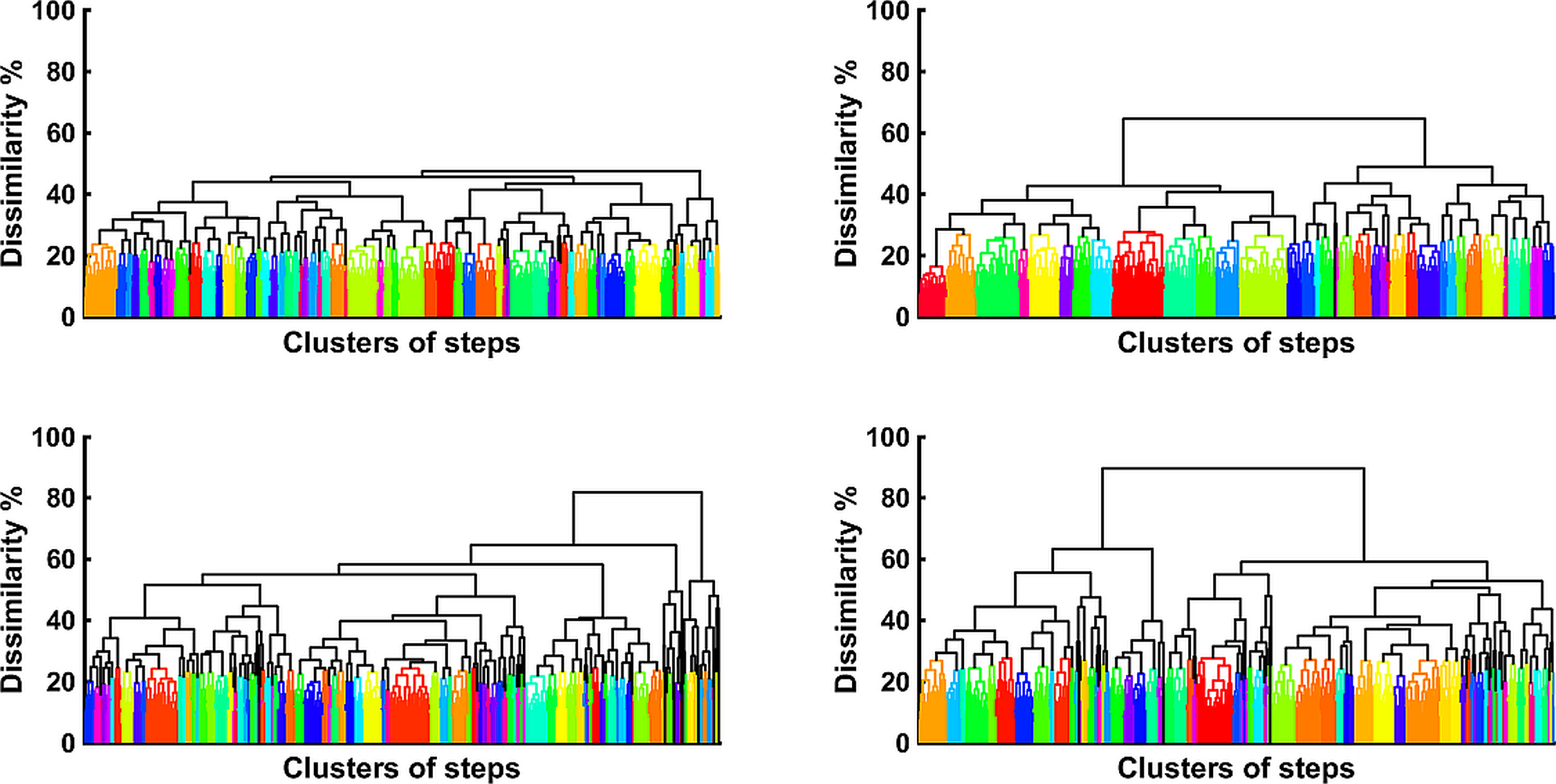

Supplement: S1 Fig — Hierarchical clustering of the intensity patterns recorded from 1221 steps of subject # 4. VM (Top left) and VL (Top right) and for 1221 surrogate patterns of VM (Bottom left) and of VL (Bottom right). (TIF) [file pone.0195125.s002.tif]
